# Use of Ultraviolet C-Radiation Therapy in Trauma and Orthopaedics: A Current State of Evidence

**DOI:** 10.7759/cureus.101151

**Published:** 2026-01-09

**Authors:** Vasileios P Giannoudis, Samuel W King, Hannah Matthews, Paul L Rodham, Oliver Vickers, Hemant Pandit, Bernard Van Duren

**Affiliations:** 1 Trauma and Orthopaedics, Leeds Teaching Hospitals Trust, Leeds, GBR; 2 Trauma and Orthopaedics, University of Leeds, Leeds, GBR; 3 Trauma and Orthopaedics, Huddersfield Royal Infirmary, Huddersfield, GBR; 4 Leeds Orthopaedic and Trauma Sciences, School of Medicine, University of Leeds, Leeds, GBR; 5 Leeds Institute of Rheumatic and Musculoskeletal Medicine, University of Leeds, Leeds, GBR; 6 Hip and Knee Arthroplasty Surgery, Nottingham Elective Orthopaedic Service, Nottingham, GBR

**Keywords:** arthroplasty, infection, sterilisation, trauma, ultraviolet-band c

## Abstract

Ultraviolet-C (UV-C) radiation exhibits potent germicidal capabilities through DNA and RNA damage in microorganisms. Its utilisation in orthopaedics began primarily for theatre sterilisation. Current applications focus extensively on surface, air, and equipment sterilisation within orthopaedic theatres. The aim of this scoping review was to establish the current uses and practices of UV-C in orthopaedic surgery.

A comprehensive literature review was conducted according to PRISMA guidelines, sourcing articles from PubMed/MEDLINE and Google Scholar from inception till January 2025. Studies included addressed UV-C applications in orthopaedic sterilisation and disinfection, financial analyses, and clinical infection outcomes. Data extracted included study parameters, methods, UV-C exposure specifics, microbial colony counts, and outcomes.

Out of 2574 articles identified initially, 23 studies met the inclusion criteria. Fifteen articles addressed UV-C sterilisation, highlighting substantial microbial reductions on theatre surfaces and equipment, although effectiveness varied by organism and exposure duration. Eight studies investigated UV-C’s impact on infection rates and skin health, showing significant infection reduction in arthroplasty cases without major adverse dermatological effects. Financial analysis was available from only one study, which indicated significant cost savings compared to traditional methods.

UV-C radiation appears effective in reducing microbial contamination in orthopaedic theatres, complementing traditional cleaning practices. It offers promising infection reduction in arthroplasty with manageable safety concerns. Indeed, use of UV-C may be an efficient way of reducing infection risk without the need for antibiotic use, and this can contribute to reduction in the risk of antimicrobial resistance. Future research should focus on refining protocols and exploring broader clinical and economic impacts.

## Introduction and background

Surgical site infections (SSIs) remain among the most common post-operative complications. In orthopaedic surgery, often due to the underlying soft tissue injuries and use of metallic implants, there is a higher associated risk. A generally accepted incidence of SSIs varies between 2 and 5%. One subset of SSIs within arthroplasty is periprosthetic joint infections (PJIs), which occur in 0.5-2% of primary hip/knee arthroplasty cases [1,2] but may rise to 10% in revision procedures. The economic impact of SSIs/PJIs globally is difficult to quantify, but estimates have the projected economic burden of PJIs in hip/knee arthroplasty to be $1.85 billion by 2030 [3]. In addition to this, a further subset of SSIs are fracture-related infections (FRIs), which occur in the context of a fractured bone, usually associated with the implantation of metalwork. The incidence varies significantly between closed (1-2%) and open (~30%) fractures [4].

Despite decades of research in addressing the issues of PJIs/ SSIs, the incidence of these conditions has remained static. Ultimately, the lack of progress emphasises the importance of prevention rather than cure.

One of the peri-operative strategies in the prevention of PJI/SSI/FRI is ensuring adequate theatre sterility of both the operating room and the equipment used. Currently, most theatre sterilisation regimes are reliant on alcohol-based theatre cleaning agents [5]. Theatres are manually cleaned prior to and after each case and at the end of the working day. This is resource-intensive, and traditional manual theatre cleaning can introduce human error. 

In addition, a universally recognised issue is anti-microbial resistance (AMR) when attempting to treat surgical infections [6-8]. For example, multiple studies have investigated the use of peri-operative single-dose antibiotics vs multiple doses to assess differences in patient outcomes and prevention of AMR [9]. Identifying solutions in controlling infection without the use of antibiotics is globally imperative [10].

An adjunct that can be used to achieve both theatre sterilisation and reduce microbial load without the presence of antibiotics is ultraviolet-C (UV-C) radiation.

UV-C is part of the ultraviolet spectrum (100-280 nanometres), which was discovered in the late 19th century [11]. The germicidal properties were recognised in 1878 and Downes and Blunt proposed its use to sterilise areas and reduce the spread of pathogens [12]. Due to these properties, its use expanded into various industries, including medicine. The mechanism of UV-C inactivation of microorganisms is to damage the genetic material in the nucleus of the cell or nucleic acids in the virus [13]. The UV-C spectrum, especially the range of 250-270 nm, is strongly absorbed by the nucleic acids of a microorganism and, therefore, is the most lethal range of wavelengths for microorganisms. This range, with 262 nm being the peak germicidal wavelength, is known as the germicidal spectrum [14]. The light-induced damage to the DNA and RNA of a microorganism is often the result of pyrimidine molecule dimerisation. Thymine is found only in DNA and its exposure to UV-C produces cyclobutane dimers. This dimerisation often prevents nucleic acid replication or makes it highly defective, preventing microorganism viability [15].

In orthopaedics, UV-C radiation was first used in 1937 at Duke University by Hart et al. (USA) as part of the sterilisation process in theatres [16]. To date, UV-C radiation has been employed primarily as a disinfection and sterilisation tool in orthopaedics [17]. It has most commonly been used in arthroplasty theatres, where its role in eliminating pathogens from surfaces, air, and surgical tools has proven to be highly effective [18,19]. Additionally, UV-C radiation is increasingly incorporated into air purification systems and sterilisation cabinets to maintain a sterile environment [20-22]. Recent studies have highlighted the effectiveness of UV-C radiation in reducing microbial load in arthroplasty theatres, with UV-C light significantly reducing the presence of bacteria, including Staphylococcus aureus and MRSA, on surfaces and in air [23].

UV-C potentially offers a reliable and cost-effective adjunct/alternative when compared to traditional cleaning methods [24]. Studies have demonstrated that manual cleaning of high-touch surfaces near the operative field can vary significantly, with one study demonstrating that in 40% of cases, high-touch surfaces were sub-optimally cleaned in theatres [25].

UV-C requires less labour and fewer chemical cleaning agents, reducing staffing and material costs. Studies suggest that while UV-C systems have a higher upfront cost, they can reduce the frequency of surface contamination and the need for frequent chemical disinfection [26].

Despite potential benefits, UV-C has not been universally implemented in orthopaedic theatres. Of note, there are some concerns related to the use of UV-C in theatres, primarily focussing on the impact of its prolonged exposure to skin and eyes [24]. Also, whilst it has bactericidal properties, it cannot distinguish between healthy and infected cells and given its impact at a molecular level, some evidence suggests that cells can undergo neoplastic metaplasia. Therefore, in hospitals where it is used there are strict protocols in place and theatre staff must wear appropriate protective equipment to avoid skin irritation and ocular disturbances. 

The purpose of this narrative review is to summarise the evidence available assessing the effectiveness of UV-C in the orthopaedic theatre setting and highlighting any potential risks or side effects reported with its use. This includes its use in the sterilisation of devices, theatre equipment, airflow and patient skin flora.

## Review

Methods

A comprehensive search strategy was undertaken using the "Preferred Reporting Systems for Systematic Reviews and Meta-Analysis (PRISMA)" statement using the search terms “(UVC or UV-C OR Ultraviolet Band C)” and “(Arthroplasty OR Hip OR Knee OR Orthopaedics)". The search was carried out using PubMed/ MEDLINE and Google Scholar and identified from its inception to 01/01/2025. The search strategy is outlined in Appendix A.

The articles identified by the initial search were screened for relevance according to our eligibility criteria by two independent reviewers (VG, SK), and then further exclusions were made after reading the main body of the texts. Where there was disagreement about whether to include an article, a third independent reviewer (HGP) acted as an arbitrator and made the final decision.

Eligibility Criteria

Studies were included in our review if they met the following criteria: (i) Case reports, clinical trials, case series, and prospective/retrospective cohort studies analysing the use of UV-C in the orthopaedic theatre setting; (ii) Any paper describing the use of UV-C as a disinfectant in commonly used theatre equipment/uniforms (e.g. theatre shoes); (iii) Any studies which reviewed the cost analysis of implanting UV-C in orthopaedic theatres.

Exclusion Criteria

Studies were excluded from our review if they met the following criteria: (i) Studies not written in the English language; (ii) Studies not performed in human/clinical workplace settings; (iii) Studies not using UV-C; (iv) Previously published systematic reviews/narrative reviews looking at UV-C.

Assimilation of Data

Included studies were analysed and specific data points extracted included: sample size; object of sterilisation; wavelength of UV-C; duration of UV-C use; analysis of colony-forming units (CFUs) and conclusions of the study.

Results

Our search yielded 2574 articles published between the timeframe of interest. After removing duplicates and screening titles and abstracts, 26 met the inclusion criteria for further analysis. Three papers were subsequently excluded due to the focus of these studies being on the molecular effects of UV-C with no direct orthopaedic relevance [27-29]. This left a total of 23 papers for in-depth analysis of which 22 were quasi-experimental observational studies with the final one being an experimental environmental study [30]. 

**Figure 1:**
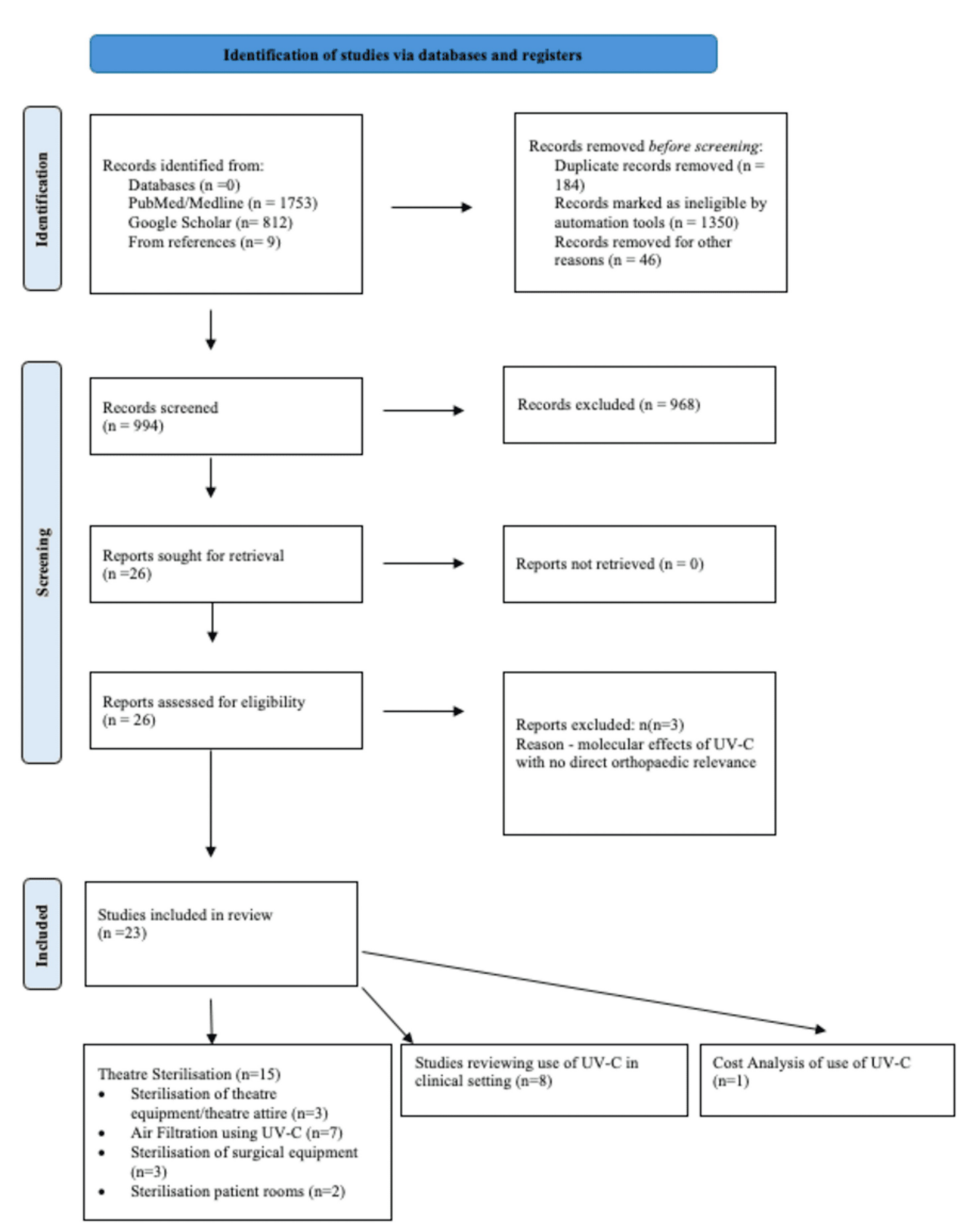
PRISMA flowchart of UV-C studies PRISMA: Preferred Reporting Systems for Systematic Reviews and Meta-Analysis; UV-C: Ultraviolet Band C Radiation.

In total, there were 15 studies which focussed on the sterilisation process using UV-C in the theatre setting. Three studies looked at the use of UV-C in sterilising clinical equipment/theatre shoe attire [31-33]. Seven studies reviewed the use of UV-C in theatres either as part of the air filtration system or alternatively in sterilising the theatre pre-/post-operatively [20,34-39]. Three studies reviewed the impact of UV-C light being placed over the surgical equipment table [21,40,41]. Finally, two studies reviewed the impact of UV-C in patient rooms as part of the sterilisation process (Table 1 and Figure 1) [42,43]. There were eight studies which reviewed the use of UV-C in the clinical setting and its effect on limiting infection or the impact on healthy skin (Table 2) [16,18,19,21,44-47].

**Table 1:** Sterilisation processes occurring using UV-C CFUs: Colony-Forming Units; HAI: Hospital-Acquired Infection; NGS: Next-Generation Sequencing; Non-HAI: Non-hospital Acquired Infection; OR: Operating Room; TPC: Total Particle Count; UV-C: Ultraviolet Band C Radiation; VPC: Viable Particle Count.

Author	Year	Equipment Sterilised	Sterilisation Device	UV Type (Wavelength)	Duration of Sterilisation	Sterilisation Outcome	Other Comments
Allen et al. [31]	2020	I-Pad	PhoneSoap, Lindon, UT, USA	UV-C (3275 μW/cm^2^)	30 seconds	UV sterilisation vs no cleaning protocol outcome in terms of CFUs OR 0.29 (CI 0.09-0.095, P=0.04)	The mean number of CFUs following cleaning with germicidal wipes was 215, compared to 233 on days utilising UV irradiation and 713 on days with no routine cleaning.
Lontano et al. [32]	2024	Healthcare Worker Smartphones	UV SANITIZE ULX – 1059 from Ulsonix	UV-C (Not stated)	6 minutes	UV sterilisation vs 70% isopropyl alcohol wipes. Immediately post intervention, the total bacterial load was almost eradicated following the use of wipes (median 1; IQR (0-5)) and UV-C boxes (median 0.5; IQR (0-2)); 3hrs post-intervention. Total bacterial load values on smartphone surfaces treated with UV-C boxes (median 22.5; IQR (10-37)) were higher than the values on smartphones sanitized with alcohol-impregnated wipes (median 10; IQR (4-23)). This indicates a greater residual effectiveness of the disinfectant in wipes, albeit at the threshold of statistical significance (P=0.056)	Swabs taken prior to sanitisation. Immediately after sanitisation treatment and 3 hours post-sanitisation process. Underpowered study. 71 workers’ smartphones were included in the study. 503 professionals per treatment arm required for appropriate powering of study.
Torres-Teran et al. [33]	2022	Theatre Crocs	HealthySole PLUS UV-C light shoe-decontamination device (HealthySole, Incline Village, NV)	UV-C (Not stated)	8 seconds	UV-C treatment significantly reduced transfer of a composite of the vegetative pathogens to floors (27 (84%) of 32 vs 9 (28%) of 32; P<0.001).	UV-C treatment did not significantly reduce transfer of C. difficile (12 (38%) of 32 versus 7 (22%) of 32; P =0.58)
Herrera et al. [35]	2024	Surgical Theatres/ ICU	UV-C light robot: ASSUM (Autonomous Sanitary Sterilisation Ultraviolet Machine).	UV-C (Peak of 254 nm by 4.25 W)	Mean (±SD) effective duration of disinfection with ASSUM was 15.11 (1.93) minutes.	Measured number of surgical site infections pre- and post-use of ASSUM. The rates of surgical site infections for all procedures in the pre- and post-intervention periods were 8.67% (80/922) and 7.5% (61/813) (p=0.37).	The dose of UV-C received at 1 meter is 1273.8 J/m^2^. Theatres were terminally disinfected with ASSUM after routine manual cleaning on the afternoon of every working day. 3/6 operating rooms were free of bacteria after manual cleaning, and all 6 were free after ASSUM operation; mean (SD) pre-ASSUM and post-ASSUM bacterial counts were 0.4 (1.2) CFU and 0 (p=0.083).
Fickenscher et al. [34]	2023	Theatre	UVHammer (Nemko, USA)	UV-C (Not stated)	Mean time of disinfection for chemical treatment was 49 minutes versus operator-driven UV emitter 7.9 minutes (P<0.001).	The operator-driven UV device outperformed chemical treatment in reducing the number of contaminated sites in the OR by more than half (P<0.001). Operator-driven UV-C reduced contaminated sites after chemical treatment by nearly half (P<0.001)	UV Hammer trainer operator device, which has a UV-C blocking shield to protect the machine handler. All theatres were initially cleaned followed by UV-C or other chemical treatments (ammonium-based).
Jones et al. [20]	2022	Theatre (Air Filtration System)	Name of portable UV device not stated. Aerobiotix Inc (Miamisburg Ohio) supplied disposable filters for use with sterilisation units.	UV-C (Not stated)	Filtration system on throughout procedure	The average contamination rate of all sites was 22 ± 1.1 CFUs/m^2^/hr in the empty OR vs. 21.3 ± 4.6 CFUs/m^2^/hr with UV units and 20.3 ± 4.9 CFUs/m^2^/hr without. Viable contaminates were found in the sterile field in 25% of UV cases vs 45% non-UV. These differences were not statistically significant.	Comparison of CFUs in orthopaedic theatres using portable UV-C filtration systems vs traditional ventilation system (non-laminar) flow. Analysis also performed comparing the number of theatre staff- (6 vs 7 staff)
Kirschman et al. [39]	2014	Theatre (Air Filtration System)	Crystalline-UV-C (C-UV-C) Air Disinfection Device (Aerobiotix, Dayton, OH)	245nm C-Band Wavelength	30 minutes	A large number of viable airborne particles were found in the active positive-pressure OR, with an average of 18628 per m^3^ in the 1.0-10.0 um diameter range. Thirty minutes after activation of the C-UV-C device, the number was reduced to 914 viable particles per m^3^ in the same size range. Reductions were consistent across the particle sizes.	Air samples were taken before and after 30 minutes of activation of the C-UV-C filtration system.
Curtis et al. [36]	2017	Theatre (Air Filtration System)	T1 C-UV-C Air disinfection-recirculation unit	UV-C (Not stated)	23 minutes (machine turned on 30 minutes before sterilisation)	Compared to controls, the cases with the C-UV-C unit at 4 m had significantly lower particle levels. Overall total particle counts (TPC)/viable particle counts (VPC), changes in TPC/VCP and maximum TPC/VPC were all significantly lower (P < .05) in the C-U-VC unit (4 m) group compared to the controls. The C-UV-C at 8 m significantly reduced TPC in all 3 outcomes (P < 0.05) compared to controls; however, it did not significantly reduce changes in VPC (P= 0.107) and maximum VPC (P= 0.052).	Study conducted to observe changes in the air particle count in theatres using UV-C. UV-C placed at 4 metres and 8 metres distance from the operative table. Particle counter measured both viable/non-viable particles in the theatre.
Fernández- Rodríguez et al. [37]	2024	Theatre (Air Filtration System)	Illuvia Sense, Aerobiotix Inc., Miamisburg, Ohio	UV-C (Not stated)	Machine turned on 30 minutes before incision	Swabs were taken intra-operatively to assess for organisms in theatre. Overall, 19/200 (9.5%) swabs isolated microorganisms. Of these, 19 (9.5%) were positive for culture, and 4 (2%) also identified microorganisms by next-generation sequencing (NGS). A wide variety of Gram +ve, Gram -ve, and anaerobic bacteria were isolated, but fungi were only recovered from inlet air swabs. The detection of microorganisms was also higher when more door openings were performed (32.5 ± 7.1 versus 27.9 ± 5.6; P < 0.01).	Study conducted to assess differences in inlet/ outlet air flow in theatres when air is sterilised through the UV air filtration system.
Anis et al. [38]	2019	Theatre (Air Filtration System)	T1 C‐UV-C disinfection and recirculation unit (Aerobiotix Inc., Dayton, OH)	UV-C (Not stated)	For half of the procedures UV-C was turned on 30 minutes prior to incision.	The C‐UV-C group had significantly lower TPC (2.6 × 10^6^ vs. 4.7 × 106 particles, p = 0.001) and VPC (18,605 vs. 27,516 particles, p = 0.001). There were fewer CFUs in the C‐UV-C group (10.9 CFU/m^3^ vs. 13.7 CFU/m^3^, p = 0.163). Multivariate analysis identified C‐UV-C filtration as a significant predictor of decreased TPC and VPC after accounting for door openings and number of OR staff.	The C‐UV-C unit was placed in the OR, 2 m from the surgical table and 8 m from the door to the sub sterile corridor. A BioTrak Real-Time Viable particle counter was used to assess the number of particles. 50 elective primary total hip/ knee procedures were performed. 25 performed with C-UV-C unit on 30 minutes prior to incision, and 25 cases randomised with unit off.
Jennings et al. [40]	2022	UV light placed over surgical equipment table	Name of UV LED emitting lamps not stated	UV-C (278nm) 5 LED lights present over table	Lights on throughout procedure	Statistically significant difference in total CFUs between the intervention vs sham at 24- hours (27 vs 95, P = .0001) and 48-hours (38 vs 122, P < .0001). The multivariate analysis revealed that the 24-hour and 48-hour count, the predictors UV light (P = .002) and hour of plate removal (P = .050) were statistically significantly associated with CFU counts.	Comparison of the UV-LED device vs Sham UV-LED device.
Carlsson et al. [21]	1986	UV light placed over surgical equipment table	UV Sterilisation light in theatres (8 Phillips TUV 40W lamps) were mounted to the ceilings.	UV-C was 25- 30 μW cm^-2 ^at level of operating table.	Lights on throughout procedure	Continuous air sampling near the wound recorded at 30-minute intervals did not demonstrate significant alterations in air contamination. The number of bacteria on the settle plates was significantly lower under UV radiation at all three sampling sites (p<0.005)	Two volumetric air samples and blood agar settling plates used from three sampling sites in theatre.
Sanzén et al. [41]	1989	UV light placed over a surgical equipment table	UV Sterilisation light in theatres (8 Phillips TUV 40W lamps) were mounted to the ceilings.	Wavelength 253.7 nm and irradiance was 25- 30 μW cm^-2^at level of operating table.	Lights on throughout procedure.	Comparison of air sampling results in 20 consecutive hip procedures. All members of staff wore occlusive clothing and UV radiation was used in half of the cases. Air sampling results taken from two regions: 1) Close to the wound; 2) In the periphery, colony-forming units taken from close to the wound. Without UV Lights on - 9.8 CFUs With UV Lights on - 2.6 CFUs P<0.001. Colony-forming units taken from the periphery. Without UV lights on – 8.6 CFUs. With UV lights on – 1.6 CFUs P<0.01	All 6 members of the operating room staff wore clean room garments (Two-piece suit with long sleeves containing a layer of expanded polytetrafluoroethylene, a material with pores about 0.2 μm (Gore-tex W.L. Gore & Associates Inc., Elkton, MD, USA). On top of the occlusive garments, the scrubbed members of the team used disposable operating gowns of nonwoven material (Molnlycke AB, Sweden) and wore double latex gloves. Safety goggles (Visorgoggles, American Ultraviolet Company) and a sun-protection cream (Eversun ultraviolet blocker) protected the eyes and uncovered skin against the ultraviolet radiation. Air sampling was conducted using the sartorius filter.
Rock et al. [42]	2018	Patient rooms in the haematologic malignancy suite	UV-C light to be used as an adjunct in cleaning rooms and bathrooms in side rooms.	UV-C (not stated)	5 minutes	Survey assessing patient and health care workers views on using UV-C technology in patient rooms as the sterilisation method. 84% said the purpose of the UV-C light was well explained, 64% had let staff know when their room was available for UV-C disinfection, 93% felt comfortable with the UV-C light operating in the bathroom while they were in the room, and 93% reported that the UV-C light did not interfere with their daily schedule. Thirty-nine percent had at some time refused UV-C light disinfection in their room or bathroom: reasons included not feeling well (25%), wanting to sleep (13%), not wanting to be bothered (11%), and not liking the smell (5%).	One hundred participants took part in the survey. Over the three days prior to the survey, UV-C light was used in patients participating room for a median of 2 times.
Rutala et al. [43]	2017	Patient rooms	UV-C light arm to be used as an adjunct to traditional cleaning techniques (ammonium-based disinfection)	UV-C (not stated)	Not stated	Study compared patient rooms with confirmed hospital acquired infections (HAI) vs non-hospital acquired infections (Non-HAI). HAI Group (Mean +/- SD) Overall CFUs 39.3 +/- 72.6 Standard Disinfection 65.1 +/- 101.9 Enhanced Disinfection 26.5 +/- 47.5 Non- HAI Group (Mean +/- SD) Overall CFUs 35.6 +/- 67.5 Standard Disinfection 35.5 +/- 61.2 Enhanced Disinfection 35.6 +/- 70.5	Enhanced disinfection may be beneficial in rooms where there are confirmed HAIs.

**Table 2:** Intra-operative use of UV-C involving human tissue (sterilisation of implants/periprosthetic joint infection) CFUs: Colony-Forming Units; CPD: Cyclobutene Pyrimidine; UV-C: Ultraviolet Band C Radiation; US: Ultrasound

Author	Year	Sterilisation Device	UV Type (Wavelength)	Duration of UV	Study participant	Infection cases	Complications	Other Comments
Hart et al. (16)	1960	UV sterilisation light in theatres (name of device in theatres not provided)	253.7- 290nm delivered with an intensity of 24-30 microwatts per cm^2^	Throughout cases in theatres.	4,585 participants. Multiple specialities (Orthopaedics, General Surgery and Neurosurgery)	Unable to ascertain the number of operative cases from 1930-1941, which were orthopaedic. However, infection rates without radiation 16.5% and with radiation 0.74%. 2/ 2600 deaths noted from infections when UV-C available to use from 1936-1946.	Erythema if exposure “greater than several hours”	17 deaths initially noted (1.3%) of patients between 1930-1936 after “clean” operations without UV. Increase in culture organisms depending on the time of year operating, number of occupants and duration of operation
Drennan Lowell et al. (44)	1980	UV Lamps	Level of intensity is 25-30 microwatts per second per square centimetre	UV lights on at the time of incision and closed at time of wound closure	Hip and Knee Arthroplasty	Hips Pre UV radiation; Primary Hips 11/519 (2.1%); Revision Hips 8/102 (7.8%); Total 19/621 (3.06%); Post UV Radiation Primary Hips 6/1516 (0.4%) Revision Hips 3/196 (1.5%); Total 9/1712 (0.53%); Knee Pre UV-Radiation Primary Knee 6/63 (9.52%); Revision Knee 1/5 (20%); Total 7/68 (10.3%); Post UV Radiation Primary Knee 4/1424 (0.28%); Revision Knee 8/100 (8%); Total 12/1524 (0.79%)	Complications secondary to UV not documented.	Floors of irradiated theatres grew no organisms during inhouse experiments. 2-3 minutes direct exposure to UV-C can lead to conjunctivitis-like symptoms. Cost of equipping 2 theatres with UV lights was $1500. Authors report cost neutral and cheaper than air filtration techniques.
Fukui et al. (45)	2020	SafeZoneUVC device (Ushio Inc. Tokyo, Japan)	222-nm. Authors used differing doses at 50/ 100 and 200 mJ/cm^2^	Not clearly stated	Healthy participants (Skin of back irradiated between T3 and T12)	The amount of cyclobutene pyrimidine (CPD) which acted as a surrogate marker of DNA damage was higher in the radiated tissue compared to irradiated tissue (p= <0.0001).	No erythema seen with 222nm and differing doses of UV-C radiation.	Follow-up of patients at three months showed no post-irradiation syndrome symptoms
Ritter et al. (18)	2007	UV Sterilisation light In theatres (name of device not provided)	UV-C (23 μW cm^-2^ and a frequency of 2537 Å)	UV lights were turned on once personnel were gowned and patient fully prepped and draped. Remained on till wound closure.	5980 joint replacements in 3846 patients. Of the 5980 total joint procedures 4909 (82.1%) performed with UV.	Hips Pre-UV Radiation; Primary Hips 4/305 (1.3%); Revision Hips 0/85 (0%); Total 4/390 (1.0%); Post UV Radiation Primary Hips 9/1258 (0.7%); Revision Hips 2/261 (0.8%) Total 11/1519 (0.7%); P values; Total p=0.5407; P values Primary p=0.3038 P values; Revision p=1.000; Knee Pre UV-Radiation Primary Knee 12/638 (1.9%); Revision Knee 3/43 (7%); Total 15/681 (2.2%); Post UV Radiation Primary Knee 17/3227 (0.5%); Revision Knee 0/163 (0%); Total 17/3390 (0.5%); P-values Total- <0.0001; P-values Primary = 0.0003; P-values Revision = 0.0086	Complications secondary to UV not documented.	The infection rate was 1.77% (19/1071) following procedures performed without the use of ultraviolet lighting and 0.57% (28/ 4909) following procedures performed with the use of ultra-violet light (p < 0.0001). Most frequently reported time to infection was 14 days, with 19% (n=9) of the 47 infections occurring at that time. 35 (74%) of the 47 infections occurred within four months.
Carlsson et al. (21)	1986	UV sterilisation light in theatres (8 Phillips TUV 40W lamps) were mounted to the ceilings.	UV-C was 25- 30 μW cm^-2 ^at level of operating table.	UV lights were on prior to incision.	30 THR procedures performed. 15 with UV and 15 without.	No infection differences noted.	No adverse effects of UV radiation noted. Wound was inspected at seven days post-operatively and no reddening of the surrounding skin noted. All wounds healed uneventfully.	To protect patients’ eyes from UV radiation, goggles were worn.
Moggio et al. (19)	1979	UV Sterilisation lights in two theatres. One theatre provided 8 unshielded UV ceiling lights (Theatre A) whereas the other had UV lights installed within ceiling pans (Theatre B)	UV-C was 17 μW cm^-2 ^in one theatre (Theatre A) and 12 μW cm^-2 ^in the other theatre (Theatre B)	UV-C on for duration of procedure. The lead surgeon decided which cases UV-C used.	47 THR procedures performed. 28 performed with UV-C and 19 without.	Theatre A – THA Cases n=23 Average number of CFUs- 1.9 Theatre B- THA Cases n=24 Average number of CFUs- 2.6 UV Lights on- CFUs 1.4 UV Lights off- CFUs 3.2 Unable to ascertain if differences in CFU between the theatres with subset analysis of UV-C being on/ off compared to differing intensity of UV-C; Surgeons present results from large retrospective case series; infection rate of 0.15% for 1322 THRs. They do not present results directly relating to the 47 THRs.	No adverse effects of UV-C reported in the study.	Surgeons investigated which infections were airborne-related and which were ‘autoinfection’ cases. Installation of the UV-C system is relatively inexpensive to install and maintain.
Nussbaum et al. (47)	1994	Birtcher cold-quartz lamp	UV-C (250nm)	15 seconds	Treatment of pressure ulcers in patients with spinal cord injuries (20 patients with 22 pressure wounds)	Comparison of UV-C in combination with ultrasound therapy vs laser treatment vs control. Percentage changes in ulcer sizes: Control 32.4% change in size US/UV-C 53.5% change in size laser 23.7% change in size	No adverse effects were reported in the US/ UV-C group. Two patients from the control group were admitted to the elective spinal centre for surgical debridement of their ulcers.	The laser protocol consisted of three treatments weekly using a cluster probe with a n 820-nm laser diode and 30 super luminous diodes (10 each at 660, 880, and 950 nm), an energy density of 4 J/cm^2^ and a pulse repetition rate of 5,000 pulses per second. The U S/W C regimen consisted of five treatments weekly, alternating the treatment modality daily. The pulsed US was applied at a frequency of 3 M H z and a spatial average-temporal average intensity of 0.2 W/cm^2^ (1:4 pulse ratio) for 5 minutes per 5 cm^2 ^of wound area.
Zhang et al. (46)	2023	ZYY-9 ultraviolet therapeutic apparatus	UV-C (254nm)	The probe of ultraviolet therapeutic apparatus was put at 5 cm from the skin. The first biological dose of ultraviolet light was 10 (20 s) per day. The dose decreased by 1 dose per day. The treatment lasted for 3 days.	Treatment of oozing wound 26 days post operative tibial plateau infection.	Biochemically, the patient was noted to have elevated markers despite wound swabs not growing organisms presumed to be infective case. Patients were given IV vancomycin.	No adverse effects were reported.	During 3-day consecutive UV-C treatment, the yellowish wound exudate gradually reduced and dis- appeared. Subsequently, the non-healing postoperative wound scarred over and healed rapidly. UV-C can promote rapid healing of recalcitrant wound disunion caused by implants in complex tibial plateau fracture.

Finally, there was one study which reviewed the financial impact of UV-C vs other conventional sterility methods intra-operatively (Table 3) [22].

**Table 3:** Comparison of cost effectiveness of UV-C CH: Charnley Howorth Enclosure; UV-C: Ultraviolet Band C Radiation.

Author	Year	Sterilisation Device	UV Type (Frequency)	Duration of UV	Study comparator	Cost Comparison	Other Comments
Berg-Périer et al. (22)	1992	UV sterilisation light in theatres (20 Phillips TUV 40W lamp tubes) were mounted to the ceilings	254nm delivered	Throughout cases in theatres.	UV-C vs Charnley- Howorth Helmet (CH)	Average Cost of using UV-C in theatre $9.40 Average Cost of using Charnley Howorth Helmet $320	The bacteriological comparison recently carried out shows that UV-C can provide ultra-clean air (<10 CFU/m^3^) and that UV-C in this study was more efficient than the CH enclosure

Based on these findings the review has been split into the following themes: (i) Sterilisation of Clinical Equipment or Clothing; (ii) Theatre Air Filtration Systems; (iii) Use of UV-C over Surgical Equipment/surgical fields; (iv) Sterilisation of Patient Rooms using UV-C; (v) Cost Analysis of Implementation of UV-C.

Sterilisation of Clinical Equipment or Clothing

Allen et al. reported a statistically significant reduction in microbial CFUs on clinical devices such as tablet devices following UV-C sterilisation compared to no cleaning protocols (Odds Ratio 0.29, CI 0.09-0.095, P=0.04). Despite this positive outcome, microbial contamination was slightly higher following UV-C irradiation (233 CFUs) than those observed after using germicidal wipes (215 CFUs). However, both methods demonstrated notable superiority to no cleaning (713 CFUs) [31].

Lontano et al. assessed UV-C sterilisation in healthcare environments, comparing UV-C boxes with 70% isopropyl alcohol wipes on healthcare workers' smartphones. Both sterilisation methods achieved comparable immediate bacterial reductions, with median bacterial loads of 0.5 for UV-C boxes and 1 for alcohol wipes. However, the study indicated that after three hours, the residual bacterial load was notably higher for UV-C-treated smartphones (median 22.5, interquartile range IQR 10-37) compared to those treated with alcohol wipes (median 10, IQR 4-23), highlighting superior residual effectiveness of chemical disinfectants. However, this finding was technically not statistically significant (P=0.056), and the study was also underpowered, involving only 71 participants per treatment group compared to the recommended 503 participants [32].

Duration of sterilisation exposure also varied significantly across studies. Short exposures, such as the eight-second duration employed by Torres-Teran et al. for disinfecting theatre footwear, demonstrated substantial efficacy against vegetative bacterial pathogens, significantly reducing microbial transfer rates from footwear to floors (84% to 28%, P<0.001) [33]. Nevertheless, this brief duration proved inadequate against more resilient organisms such as Clostridium difficile, where the reduction was not statistically significant (38% versus 22%, P=0.58). Conversely, longer durations of UV-C exposure (e.g. 30 seconds and 6 minutes) were used in other studies but analysis of the specific pathogens remaining present despite the use of UV-C wasn’t performed [31,32]. The study by Allen et al. compared the use of 30 second I-pad sterilisation in a boxed UV-C device versus routine cleaning using quaternary ammonium and isopropyl alcohol germicidal wipes against a control (no formal sterilisation). Germicidal wipes were associated with a 69.9% decrease in bacteria compared with no routine cleaning, and UV irradiation was associated with a 67.3% decrease in bacteria compared with no routine cleaning. Of note. no MRSA colonies were found but five iPads were left colonised with MRSA. It is not clear whether these were in the control/ UV-C or alcoholic sterilisation wipes [31]. A similar study was performed by Lontano et al. this time focussing on healthcare professionals’ phones comparing UV-C with alcoholic wipes, with swabs being taken pre-sterilisation, at five minutes post sterilisation and three hours post sterilisation. The five minutes post sterilisation swabs taken showed almost complete eradication of bacteria irrespective of use of UV-C versus wipes. Specifically, 3 h after sanitisation, the total bacterial load values on smartphone surfaces treated with UV-C boxes (median 22.5; IQR (10-37)) were higher than the values on smartphones sanitised with alcohol-impregnated wipes (median 10; IQR (4-23)). These findings did not reach statistical significance (p=0.056) and the study was significantly underpowered. Of note the swabs which were cultured in this study were trying to isolate MRSA, VRE or CHROMID. MRSA was isolated in one phone prior to sterilisation with alcohol wipes which was subsequently eradicated in further swabs taken at five minutes and three hours. However, one phone which was in the alcohol sterilisation group which initially had no MRSA on subsequent cultures at three hours MRSA was isolated [32].

Theatre Air Filtration System

Recent studies investigating the efficacy of UV-C air filtration systems in operating theatres have demonstrated variable results concerning air sterility and reduction in microbial contaminants. Kirschman et al. documented a specific UV-C wavelength of 245 nm (C-band), as indicated by [39]. However, other studies including those by Jones et al. [20], Curtis et al. [36], Fernández-Rodríguez et al. [37], and Anis et al. [38] did not provide the precise UV-C wavelength used.

The duration of UV-C sterilisation varied among studies, ranging from the device being operational throughout the procedure [20] to a consistent practice of activating the device approximately 30 minutes prior to surgical incision [36-38] or sterilisation assessments [39].

In terms of efficacy, studies collectively suggest a positive trend in reducing total and viable airborne particles, enhancing sterility within theatres. Kirschman et al. reported a substantial reduction from an average of 18,628 to 914 viable particles per cubic meter within 30 minutes [39]. Similarly, Curtis et al. observed significant reductions in particle counts at 4 m from the surgical field, with a reduced effect at 8 m [36]. Anis et al. found significant reductions in total and viable particle counts using UV-C filtration; however, the decrease in CFUs was not statistically significant [38]. Jones et al. noted fewer viable contaminants in UV-treated theatres (25%) compared to non-UV-treated environments (45%), yet these differences were not statistically significant [20]. Fernández-Rodríguez et al. identified microorganisms in 9.5% of theatre swabs, suggesting effectiveness yet highlighting the continued presence of contaminants, particularly linked to increased door openings [37].

Overall, UV-C air filtration systems, predominantly activated for about 30 minutes pre-procedure, demonstrate a promising but heterogeneous effect on enhancing sterility in operating theatres, necessitating further research to standardise protocols and evaluate long-term clinical outcomes.

Use of UV-C Over Surgical Equipment/Surgical Fields

UV-C irradiation demonstrates significant efficacy in sterilising surgical equipment within operating theatres, as evidenced by multiple studies. Jennings et al. employed UV-C LEDs (278 nm wavelength) continuously during surgical procedures, identifying a statistically significant reduction in colony-forming units (CFUs) on equipment at both 24 hours (27 CFUs intervention vs. 95 CFUs sham; p=0.0001) and 48 hours post-procedure (38 CFUs intervention vs. 122 CFUs sham; p<0.0001). This highlights UV-C's effectiveness in maintaining sterility over an extended period [40].

Earlier studies corroborate these findings. Carlsson et al. utilised ceiling-mounted Philips TUV 40W lamps delivering UV-C at 25-30 μW/cm² at the operating table, continuously during surgeries. While volumetric air samples showed no significant differences, settle plates indicated substantially reduced bacterial contamination at all sampling points under UV-C exposure (p<0.005) [21].

Similarly, Sanzén et al., employing similar Philips TUV 40W lamps (253.7 nm), consistently demonstrated significantly lower CFU counts with UV-C activation both near surgical fields (2.6 CFUs with UV vs. 9.8 CFUs without UV; p<0.001) and peripheral theatre regions (1.6 CFUs with UV vs. 8.6 CFUs without UV; p<0.01) [41]. Notably, staff adhered strictly to comprehensive protective protocols, including occlusive garments, double gloves, hoods, and protective eyewear, highlighting the additional safety considerations required when utilising continuous UV-C irradiation.

Collectively, these studies affirm UV-C irradiation as an effective adjunctive method for sterilising surgical equipment and reducing environmental contamination in operating theatres. Optimal sterilisation outcomes necessitate continuous UV-C exposure throughout surgical procedures, typically delivered at wavelengths around 254-278 nm, and with appropriate protective measures for theatre personnel to mitigate potential UV-C-related hazards.

Sterilisation of Patient Rooms Using UV-C

The impact of UV-C sterilisation technology on patient room sterilisation has been assessed in recent studies. Rock et al. evaluated UV-C as an adjunctive measure for sterilising patient rooms and bathrooms within a haematologic malignancy suite. UV-C was applied for a brief duration of five minutes per session. A survey conducted with 100 participants revealed positive acceptance of UV-C technology, with 84% indicating that the purpose of UV-C was clearly explained to them, 93% comfortable with its use in bathrooms during their stay, and another 93% reporting minimal interference with their daily routines. However, patient refusal for UV-C application was also noted, predominantly due to feeling unwell (25%), desire for sleep (13%), inconvenience (11%), and displeasure regarding odour (5%). The median frequency of UV-C disinfection per patient room was twice over three days, highlighting brief yet frequent sterilisation cycles [42].

Rutala et al. (2017) investigated UV-C technology combined with traditional ammonium-based cleaning methods, comparing rooms occupied by patients with hospital-acquired infections (HAIs) against those without HAIs. Although specific UV-C frequency and duration were not detailed, findings indicated a marked reduction in microbial CFUs in rooms undergoing enhanced UV-C disinfection, especially significant in the HAI group. Standard disinfection yielded higher CFUs (65.1 ± 101.9) compared to enhanced disinfection (26.5 ± 47.5), suggesting that enhanced UV-C disinfection is particularly beneficial in rooms associated with confirmed HAIs [43].

Both studies highlight UV-C's efficacy as an adjunct sterilisation method, demonstrating notable patient acceptance and effectiveness in microbial reduction, particularly in high-risk healthcare environments. The brevity and frequency of UV-C applications are practical considerations for broader clinical adoption.

Impact of UV-C on PJI/Human Interventions

Following the inception of UV-C in theatres, several studies have demonstrated a reduction in the number of infections in both the primary and revision arthroplasty setting. Of note, the literature which has been published is predominantly from the 20th century suggesting that there may be some ongoing reluctant to use UV-C as part of the sterilisation process.

Hart et al. was the first study to demonstrate a significant reduction in infection rates from 1930-1941 when comparing the use of UV-C (0.74%) to the absence of it (16.5%). However, based on the presentation of data, it was not possible to ascertain how many were orthopaedic cases. They also mentioned erythema developing on patients and staff skin if exposure was “greater than several hours” [16].

Two studies presented data for the outcomes of arthroplasty in both the primary and revision arthroplasty setting [18,44]. Combining the data sets prior to UV, the incidence of infection in primary THA was 1.8% (15/824) whilst in the revision (8/187) THA was 4.3%. Following the implementation of UV-C (ceiling mounted UV-C lamps were inserted to produce an intensity radiation level at the operating room as per the Hart Criteria [16]), incidence of infections decreased in both the primary THA (0.5%, 15/2774) and revision THA (1.1%, 5/457). The incidence of infection in primary TKA prior to UV-C was 2.6% (18/701) whilst in revision TKA it was 8.3% (4/48). Similarly following the introduction of UV-C there was also a reduction in incidence of infection in the primary TKA group 0.5% (21/4651) and the revision TKA group 3% (8/263). In both these studies the UV-C was on from the time of skin incision to skin closure. Neither of these studies documented any complications to patients secondary to the use of UV-C. Lowell et al. highlighted the risk of developing ‘conjunctivitis’ type symptoms to eyes if direct exposure for 2-3 minutes without protective eyewear [44]. 

Given the concerns regarding the dermatological manifestations of UV-C Fukui et al. irradiated healthy participants’ skins on their back with differing doses of UV-C [45]. The higher doses of UV-C produced greater amounts of cyclobutene pyrimidine (CPD) which acted as a surrogate marker of DNA damage. However, when following up patients at three months none of them showed any evidence of post-irradiation syndrome.

In the trauma setting, one case study showed the benefits of UV-C in managing wound complications following fixation of tibial plateau fractures. At day 26 post-operatively, the wound was noted to be producing a yellow exudate. Dual treatment with IV vancomycin and three days of UV-C treatment applied 5cm away from the wound was used. Following this, it was noted that the wound had healed rapidly, and the authors recommended its use for ‘recalcitrant wound disunion’ [46].

Cost Analysis of Implementation Of UV-C

Berg-Perier et al. presented one study comparing the cost of using UV sterilisation theatre lights vs the Charnley Howorth Helmet (CHH) per case. Based on their studies it was found that it was significantly cheaper using UV $9.4 in 1992 ($21.50 in 2025 USD) compared to use of the Charnley-Howorth Helmets $320 in 1992 ($733 in USD). Of note, bacteriological analysis also suggested that the UV-C provided cleaner air than that in the CHH enclosure [22].

Discussion

From the paucity of literature available in the use of UV-C in orthopaedics, this review has highlighted that this adjunct may potentially play a role in both sterilisation of theatres and prevention of FRI/ PJI. Studies have shown its ability in reducing CFUs when implemented as part of an air filtration system [20,36-39] and clinical studies have shown the reduction of incidence of PJIs in orthopaedics [18,44]. In addition to this, they have shown that it can also be used as a disinfectant in patient rooms [42,43], which may suggest that patients who are undergoing revision surgery or are high-risk patients for infections should have their hospital room sterilised with UV-C regularly.

Whilst there have been a multitude of studies published aimed at providing treatment strategies to manage PJIs and FRIs ultimately the mantra in orthopaedics remains that “prevention is more important than cure”. This is especially pertinent in the elective orthopaedic setting where pre-operative optimisation relating to patient modifiable factors such as glycaemic control and smoking cession is feasible. Similarly, non-patient modifiable factors like theatre set up e.g. presence of laminar air flow plays a role in reducing the incidence of infections [48]. However, in the recent International Joint Centre consensus meeting in Istanbul, experts in the field suggested that there was no evidence to demonstrate laminar flows use as a tool in effectively reducing infection in PJI or FRI. These contrasting messages again highlight the need for development of further technologies in prevention of PJI. Interestingly, during the consensus meeting, UV-C was not discussed in the prevention/ treatment in formation of a biofilm.

UV-C radiation, characterised by wavelengths ranging primarily from 250 to 270 nm and peaking around 262 nm, has gained considerable attention for its germicidal properties. The mechanism underlying its effectiveness involves the formation of cyclobutane pyrimidine dimers in microbial DNA and RNA, significantly hindering replication capabilities [49,50]. The cytotoxic capacities possessed provide the opportunity to destroy cells but the UV-C cannot discriminate between healthy and infected cells [51,52].

Historically, UV-C radiation was first utilised in orthopaedic theatres in 1937, initially aimed at sterilising the theatre environment to mitigate infection risks [16]. Since then, advancements have expanded its usage, particularly in orthopaedic surgery, to disinfect air, surgical instruments, theatre equipment, and patient room environments [20,21,34,36-41]. Contemporary research consistently highlights its efficacy against challenging pathogens, including Methicillin-resistant Staphylococcus aureus (MRSA) [53,54], demonstrating marked microbial load reductions on both air and surfaces within operating theatres. Interestingly its clinical uses in managing/preventing PJI or FRI have been sparingly investigated for over a decade. 

The reviewed literature suggests that UV-C radiation's role as an adjunct rather than a replacement for traditional manual cleaning methods. Its ability to disinfect inaccessible or difficult-to-clean regions, such as ventilation systems and equipment surfaces, substantially enhances traditional cleaning methods [34,36,39].

Economic analyses suggest long-term cost-effectiveness associated with UV-C systems [22]. While upfront costs can be substantial, the technology often reduces reliance on chemical disinfectants and manual labour, leading to potential savings over extended periods. Berg-Perier et al. specifically documented notable cost savings using UV-C compared to traditional sterile methods, indicating a significant economic advantage when UV-C systems were appropriately integrated into surgical practices [22].

Clinical outcomes from historical and contemporary studies further underscore UV-C’s impact in reducing infection rates. Notably, landmark research by Hart et al. from the early 20th century reported significant declines in infection rates in surgical settings where UV-C was employed, reducing infections dramatically from 16.5% to 0.74% [16]. Subsequent studies involving primary and revision arthroplasties, including extensive datasets presented by Lowell et al. and Ritter et al., consistently showed reduced infection incidences post-implementation of UV-C sterilisation [18,44]. For example, infection rates in primary THA decreased notably from 1.82% pre-UV-C to 0.54% post-UV-C, with similar significant reductions observed in revision THA, primary TKA, and revision TKA scenarios.

The role of UV-C in preventing the risk of AMR also warrants further investigations. Multiple in vivo studies have shown that UV-C can inactivate resistant organisms such as methicillin-resistant strain of Staphylococcus aureus and vancomycin-resistant E. faecalis (VRE) [55,56]. However, there is a paucity in the literature investigating its use in animal/human models.

Safety concerns related to UV-C are a potential limitation to regular implementation. There are potential harms associated with prolonged exposure, including skin erythema, dryness, and ocular complications. A study by Fukui et al. demonstrated minimal dermatological consequences from controlled UV-C exposure in clinical settings [45]. Precautions, such as wearing protective clothing, gloves, and eyewear, effectively mitigate these risks. Additionally, preliminary evidence supports beneficial wound healing effects from controlled UV-C exposure, warranting further investigation, particularly in infected wound management [46,47].

Despite these encouraging findings, variability in study designs and the absence of universally standardised protocols underscore the necessity for additional rigorous research. On account of this, performing a meta-analysis of results available was not feasible and therefore the stated results of these studies should be taken with caution. Future investigations should focus on optimising UV-C exposure parameters, standardising safety protocols, and conducting long-term clinical outcome analyses to enhance evidence-based integration of UV-C technology in orthopaedic surgical environments. One of the limitations of this review was the lack of blinding of reviewers to authors, journals or institutions.

## Conclusions

In conclusion, current evidence suggests that UV-C radiation may have a role in reducing microbial contamination and infection rates within orthopaedic surgical settings. Although challenges regarding safety and efficacy variability exist, its complementary role alongside traditional cleaning methods presents a promising avenue for enhancing surgical sterility and patient outcomes. Further studies with standardised protocols are required to provide higher level of evidence to analyse its use.

## Appendices

Appendix A

**Table 4:** Search strategy “OR” function retrieves any of the words included in specific strategy broadening search results. (e.g. will retrieve any article related to UV-C in the 4th line of search strategy including search terms 1, 2 and 3). “AND” function combines different concepts and both concepts must be present in search results (e.g. UV-C and differing orthopaedic themes).

MEDLINE 01 January 2025	Google Scholar 01 January 2025
1. Ultra-Violet Radiation Band C	1. Ultra-Violet Radiation Band C
2. UV-C	2. UV-C
3. Ultra-Violet	3. Ultra-Violet
4. 1 or 2 or 3	4. 1 or 2 or 3
5. Arthroplasty	5. Arthroplasty
6. Orthopaedics	6. Orthopaedics
7. Prosthetic Joint	7. Prosthetic Joint
8. 5 or 6 or 7	8. 5 or 6 or 7
9. 4 and 8	9. 4 and 8
